# Optimized Method for Efficient DNA Extraction from Agricultural Soils

**DOI:** 10.3390/mps9010024

**Published:** 2026-02-09

**Authors:** Elías Hernández-Cruz, Lorena Jacqueline Gómez-Godínez, José Martín Ruvalcaba-Gómez, Ramón Ignacio Arteaga-Garibay

**Affiliations:** Centro Nacional de Recursos Genéticos, Instituto Nacional de Investigaciones Forestales, Agrícolas y Pecuarias, Boulevard de la Biodiversidad 400, Rancho las Cruces, Tepatitlán de Morelos 47600, Jalisco, Mexico; eliashernandezcruz45@gmail.com (E.H.-C.); gomez.lorena@inifap.gob.mx (L.J.G.-G.); ruvalcaba.josemartin@inifap.gob.mx (J.M.R.-G.)

**Keywords:** DNA extraction, inhibitory compounds, prewashes

## Abstract

Soil harbors the highest concentration of microorganisms in ecosystems, and their molecular characterization through high-throughput sequencing is essential for metagenomic studies. However, obtaining high-quality, high-concentration DNA is limited by physicochemical properties (pH, heavy metals, humic acids) and adsorption to clay minerals. Although standardized commercial protocols exist, they present variable limitations depending on soil type. This study developed and validated the National Center for Genetic Resources—Microorganism Collection (CNRG-CM) method, which incorporates innovative pre-washing steps using phosphate-buffered saline (PBS) and sodium phosphate to effectively remove inhibitory humic acids and metal ions, combined with cetyltrimethylammonium bromide (CTAB)/chloroform extraction to achieve high-molecular-weight metagenomic DNA isolation. The CNRG-CM method was applied to three diverse soil types with variable physicochemical properties, recovering DNA concentrations ranging from 1000 to 1300 ng/μL ith a yield of 30 to 48 µg/g^−1^, significantly exceeding those obtained with a standard commercial kit with maximum DNA concentrations of 360 ng/μL and a yield of 43 µg/g^−1^. The CNRG-CM protocol is established as an effective and adaptable alternative for metagenomic DNA extraction across diverse agricultural and ecological contexts. It enables subsequent metagenomic studies of soil microbial communities.

## 1. Introduction

Soil harbors the largest number of microorganisms in terrestrial ecosystems, and understanding their composition and function is essential to understanding the role of these microbial communities in soil function and the ecosystem services they provide [1]. The study of soil microbiota is fundamental to understanding the ecological and functional dynamics of agricultural ecosystems, as soil microorganisms play key roles in biogeochemical processes, soil fertility, and plant health [2]. The molecular characterization of these microbial communities requires obtaining high-quality, high-concentration DNA from soil samples, which presents a significant challenge due to the complexity and heterogeneity of the substrate, as well as the presence of inhibitory compounds that hinder extraction and subsequent genomic analysis [3]. Due to the difficulties associated with culturing most soil microorganisms, their DNA is extracted directly from the soil to examine their distribution and functions in the environment [4]. In addition to the extraction challenges, the reduction in the cost of massive sequencing has enabled new projects to understand microbial ecosystems [5].

Studying the soil microbiome remains challenging, as purifying DNA extracts suitable for amplicon sequencing can be hampered by the co-extraction of secondary metabolites that inhibit enzymes, such as humic substances or fulvic acids present in soil [6]. Over time, DNA extraction has become established as a critical step for the success of a wide variety of molecular applications, especially in the field of next-generation sequencing, since the quality, integrity, and quantity of the extracted DNA directly determine the reliability of the data generated in genomic studies [7,8]. Although a wide variety of standardized and rapid DNA extraction kits are available on the market, using silica or ion-exchange columns and different buffers for soil inhibitors, their effectiveness remains limited by the physicochemical properties of the sample, which range from acidic to alkaline soils, and include high levels of heavy metals or humic and fulvic acids [8,9,10,11]. Another reason for low DNA extraction yields is that nucleic acids can be adsorbed by soil compounds such as clay, which, combined with the presence of bacteria resistant to lysis, compromises the efficiency of DNA extraction [1].

DNA extraction begins with cell lysis, a critical step that combines mechanical, chemical, and enzymatic strategies to maximize nucleic acid release. Standard methods include physical disruption by grinding or glass beads, the use of detergents and chaotropic salts such as cetyltrimethylammonium bromide (CTAB), sodium dodecyl sulfate (SDS), or β-mercaptoethanol to solubilize membranes, and enzymatic digestion with proteinase K or lysozyme to degrade the protein matrix—strategies that are frequently integrated to overcome the recalcitrance of certain soil microorganisms [12,13,14]. Following lysis, protein and lipid separation is commonly performed by phenol-chloroform extraction or chloroform alone. This method takes advantage of the differential solubility of biomolecules: phenol denatures proteins, and chloroform helps in phase separation by trapping them in the organic interface or interphase, while nucleic acids remain in the upper aqueous phase, allowing their recovery with high purity [15,16].

Once the aqueous phase is recovered, DNA precipitation is performed by adding absolute alcohol (ethanol or isopropanol) in the presence of cationic salts. These characteristics allow DNA, despite its negative charge, to precipitate by inducing its aggregation and selective insolubilization [17,18,19].

Finally, resuspension of the DNA pellet ensures its long-term stability. Where TE buffer (Tris-EDTA) or nuclease-free water is used, although TE buffer is better because it maintains an alkaline pH, EDTA acts as a chelating agent for ions that are essential cofactors for DNase activity, thus preventing enzymatic degradation of the sample [20,21]. Designing custom DNA extraction methods tailored to the nature of each sample for genomic DNA extraction remains the best option. In addition to achieving high DNA yields, it can reduce extraction costs per sample. The phenol-chloroform method has been the most widely used for decades and, when combined with chaotropic salts, glass beads, and proteases, typically yields the best results in terms of purity and DNA yield [22,23,24].

Currently, there is no universal methodology or commercial kit for nucleic acid extraction from soil samples due to the wide range of soil physicochemical properties. The objective of this work was to develop and provide an efficient protocol for extracting high-quality, high-molecular-weight metagenomic DNA from diverse soil types for future metagenomic studies.

## 2. Experimental Design

### 2.1. Sample Collection

**Figure 1:**
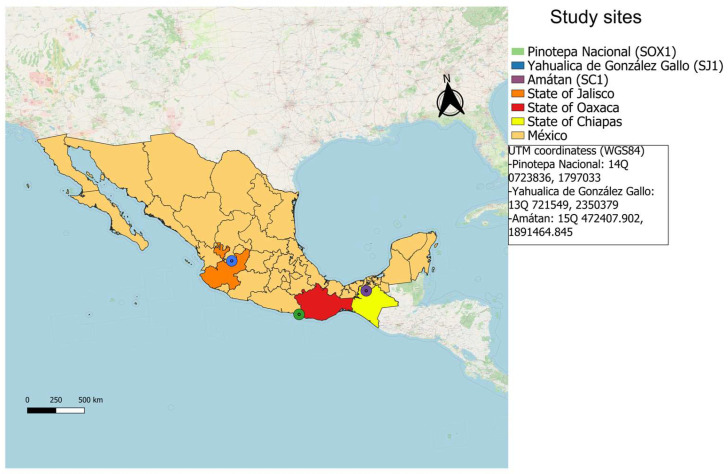
Map showing the areas where soil samples were collected in corn (SC1, SOX1) and coffee (SC2) cultivation sites. The dots indicate the municipalities where sampling was conducted.

DNA was extracted from three different soil samples collected from agricultural properties in three Mexican states (Figure 1): Amatán, Chiapas (SC1); Yahualica de González Gallo, Jalisco (SJ1); and Pinotepa Nacional, Oaxaca (SOX1). Sampling was carried out in quadrants, drilling a hole 15 to 20 cm deep with an auger, obtaining samples with a total weight of 1 kg (10 subsamples in total). The soil was placed in Ziploc bags and transported to the laboratory under refrigeration for processing.

### 2.2. Preparation and Description of Soil Samples

Once the samples arrived at the laboratory, prior to DNA extraction, soil samples were air-dried and sieved through a 2 mm mesh to homogenize the material and remove stones, roots, and visible plant debris. Soil pH was determined in a 1:2.5 (*w*/*v*) soil-to-water suspension by adding 25 mL of water to 10 g of soil. The mixture was shaken thoroughly for 60 min, allowed to stand for 60 min, and the pH was measured at 25 °C in the partially settled suspension, following the standard FAO protocol. In addition, a comprehensive description of the physical characteristics of each sample was performed, including texture [25], color, presence of visible organic matter, moisture, and structure [26,27].

## 3. Procedure

### 3.1. DNA Extraction Methods

Method 1: Modified Wilson CTAB Method [28]

Total time: approximately 3–4 h (plus overnight incubation)

Stage 1: Sample Preparation and Cell Lysis (1.5 h)

(1) Use a mortar and add 500 mg of soil, then cover with liquid nitrogen.
(2) Macerate until a fine powder is obtained, which will be placed in a 1.5 mL conical tube.
(3) Add 500 µL of Tris-HCl: EDTA (TE) buffer (1 M and 0.5 M), and shake the mixture at 3500 RPM for 5 min.
(4) Centrifuge at 12,000 RPM for 5 min and discard the supernatant.
(5) Add 500 µL of lysis buffer (Tris-HCl 1M pH8, EDTA 0.5M pH8, NaCl 2.5M, and CTAB 10%). and 5 µL of proteinase K (20 mg/mL), then stir.
(6) Incubate at 56 °C for 1 h.
(7) Shake at maximum speed for 5 min.
(8) Add 500 µL of phenol:chloroform:isoamyl alcohol (25:24:1).
  a. Stage 2: Organic Solvent Extraction—First Round (~40 min)
(9) Vortex for 3 min and centrifuge at 12,000 RPM for 10 min.
(10) Recover the aqueous phase and place it in a new tube.
(11) Add 500 µL of chloroform:isoamyl alcohol 24:1.
(12) Mix by inversion and centrifuge at 12,000 RPM for 10 min.
(13) Recover the supernatant. Optional step: Repeat this chloroform:isoamyl step one more time to improve purity.
  b. Stage 3: DNA Precipitation and Recovery (~ 2 h plus overnight incubation)
(14) Add 1 mL of cold 2-propanol to the supernatant and mix by inversion.
(15) Allow the mixture to stand overnight at −20 °C.
(16) Centrifuge at 12,000 RPM at 4 °C for 10 min.
(17) Remove and discard the supernatant.
(18) Add 1 mL of 70% ethanol and vortex.
(19) Centrifuge at 12,000 RPM for 5 min and discard the supernatant.
(20) Dry the pellet at 65 °C.
(21) Resuspend the DNA in 30 µL of nuclease-free water.

Method 2: National Center for Genetic Resources—Microorganism Collection (CNRG-CM)

Total time: approximately 4–5 h (plus overnight incubations)

Stage 1: Pre-washes (35 min)

(1) Weigh 1 g of soil into a 15 mL Falcon tube and add phosphate-buffered saline (PBS) (0.137 M NaCl, 0.0027 M KCl, 0.01 M Na_2_HPO_4_, 0.0018 M KH_2_PO_4_).
(2) Vortex at 3500 RPM for 1 min, allow to stand at room temperature for 10 min, and centrifuge at 3000× *g* for 10 min.
(3) Discard the supernatant and repeat this step twice (a total of 3 PBS washes).
(4) Add 10 mL of sodium phosphate (0.15 M Na_4_P_2_O) to the soil pellet, vortex at 3500 RPM for 1 min, allow to stand for 10 min, and centrifuge at 3000× *g* for 10 min.
(5) Repeat this sodium phosphate wash one additional time (total 2 washes).
  a. Stage 2: Chemical and Mechanical Lysis (50 min plus freeze-thaw)
(6) Add 1 mL of saline buffer (0.15 M NaCl, 0.1 M EDTA, pH 8.0) to the formed pellet, vortex, and add 500 mg of porcelain beads.
(7) Add 1 mL of lysis buffer (0.15 M NaCl, 0.6 M Tris-HCl, pH 8.0, 10% SDS), vortex, and shake at 3500 RPM for 10 min.
(8) Immediately freeze at −80 °C for one hour.
(9) Thaw at 65 °C for 30 min.
(10) Centrifuge at 4900× *g* for 20 min and recover the supernatant.
(11) Add 3500 µL of EDTA (0.5 M, pH 8) and 150 µL of potassium acetate (5 M KCH_3_CO_2_, pH 5) and allow to stand for 20 min at 4 °C.
  b. Stage 3: DNA Washes with Organic Solvents (40 min)
(12) Centrifuge at 3000× *g* for 10 min and recover the supernatant.
(13) Add an equal volume of chloroform:isoamyl alcohol (24:1) twice.
(14) Centrifuge at 3000× *g* for 10 min after each addition.
(15) Recover the supernatant and add an equal volume of polyethylene glycol (15% PEG 8000 MW dissolved in 1.6 M NaCl).
(16) Allow to stand overnight at 4 °C.
(17) Centrifuge at 3000× *g* for 10 min.
  c. Stage 4: Salt and Solvent Washes (25 min)
(18) Resuspend the pellet in 400 µL of 70% ethanol and transfer to 1.5 mL conical tubes.
(19) Centrifuge at 7000× *g* for 5 min and decant the supernatant.
(20) Add 400 µL of absolute ethanol, centrifuge at 7000× *g* for 5 min, and discard the supernatant.
(21) Allow the pellet to dry at room temperature and resuspend in 30 µL of nuclease-free water.

Method 3: Commercial Kit Protocol.

Total time: 20–30 min

Extraction Protocol.

(1) Use the Quick-DNA Fecal/Soil Microbe Kits (Zymo Research).
(2) Perform extraction according to the manufacturer’s protocol.
(3) Store at −20 °C until use.

### 3.2. DNA Quantification Obtained by Different Extraction Methods

DNA quantification was performed using aNanoDrop ND-2000, with readings 260/230 and 260/280 serving as references for nucleic acid quality and integrity. The 260/230 reading indicates DNA quality and purity, while the 260/280 reading indicates contamination by fulvic and humic acids. Humic acids and co-extracted proteins are two significant DNA contaminants from environmental samples. Humic acid and protein levels were determined by measuring absorbances at 230 nm and 280 nm, respectively, while the amount of DNA was determined by measuring absorbance at 260 nm. To confirm DNA integrity and quality, a 1.5% agarose gel was run with Red Gel at a 1:4 concentration, adding 2 µL of loading buffer and 3 μL of DNA per well. The gel was run at 90 V for 1 h, and then imaged using UV light.

### 3.3. DNA Quality Determination by PCR

A PCR assay targeting the 16S rRNA gene using universal primers 27F and 1492R was performed as a quality screening step to assess DNA integrity and amplifiability. This amplification served as an inclusion/exclusion criterion to validate samples for downstream diversity analysis by high-throughput sequencing. A 1:100 dilution of DNA extracted with the three methods was prepared to minimize potential inhibitors. The reaction mixture was prepared using the Crystal Taq kit, containing 10 μL of Taq Master Mix, 2.5 μL of primer 27F (10 μM; 5′-AGAGTTTGATCCTGGCTCAG-3′), 2.5 μL of primer 1492R (10 μM; 5′-GGTTACCTTGTTACGACTT-3′), 8 μL of PCR-grade water, and 2 μL of the diluted template DNA. Thermal cycling conditions consisted of an initial denaturation at 95 °C for 7 min, followed by 30 cycles of denaturation at 95 °C for 45 s, annealing at 56 °C for 1 min, and extension at 72 °C for 1 min 30 s, with a final extension at 72 °C for 10 min. The amplicons were run on a 1.5% agarose gel, which was run with Red Gel at a 1:4 concentration, adding 2 µL of loading buffer and 3 μL of DNA per well. The gel was run at 85 V for 1:20 h and then imaged using UV light.

## 4. Expected Results

### 4.1. Description of Soil Samples

**Figure 2:**
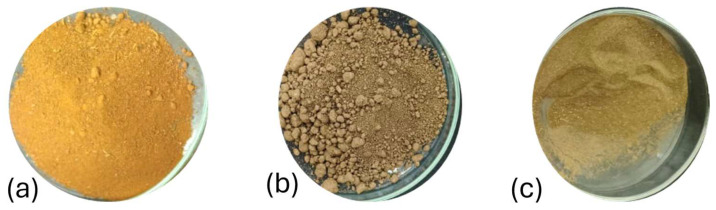
Soil samples processed in this test. Where (**a**) soil SJ1, (**b**) soil SOX1, and (**c**) soil SC1 are represented.

Three soil samples with distinct physicochemical characteristics (pH, color, and organic matter content) were used in this study, as detailed in Figure 2 and Table 1. The visual and chemical properties of soil, especially color and organic matter content, can influence the presence of inhibitors during DNA extraction and PCR amplification [29,30]. In this context, prior characterization of each sample’s physicochemical properties is essential for selecting the most appropriate extraction protocol, thereby optimizing both DNA quality and yield while minimizing bias and information loss during extraction [31,32].

The three soil samples analyzed exhibited distinct physicochemical characteristics. Sample SC1 had a silty loam texture, a pH of 6, and a dull gray color. This sample had a low organic matter content. On the other hand, sample SJ1 had a clay loam texture, a pH of 4.8, and a reddish-brown color. This sample contained a medium amount of organic matter. Finally, sample SOX1 exhibited a silty loam texture, a pH of 7.6, and a dark color. This sample stood out for its high organic matter content (Table 1).

### 4.2. DNA Extraction Using Different Methods

**Figure 3:**
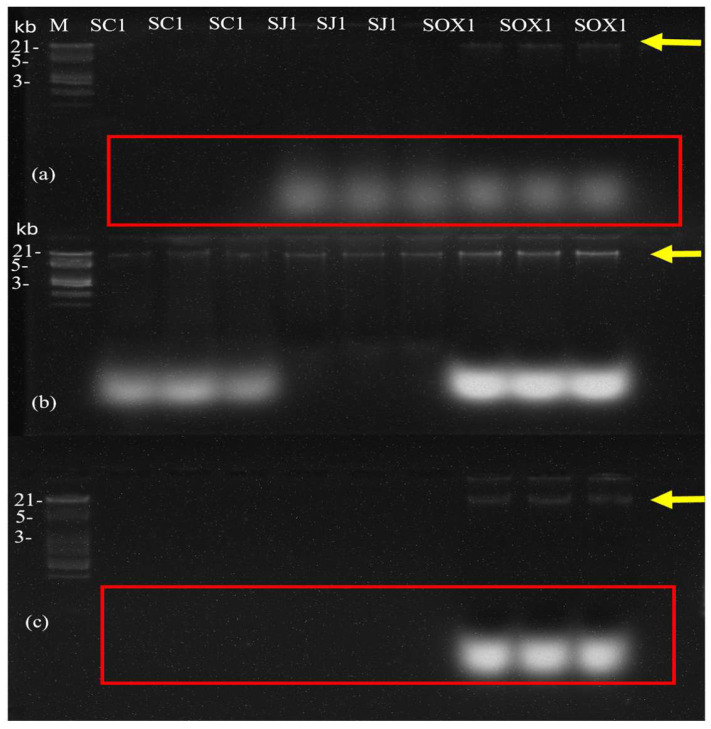
Agarose gel of genomic DNA extraction from the three soil samples in triplicate. The first lane is the lambda molecular weight marker (M). Where (**a**) CTAB method, (**b**) CNRG-CM method, (**c**) Zymo Research kit method. Photographed with 30 s exposure. Red boxes indicate probable contaminants, RNA, or residual proteins carried over during extraction. Yellow arrows indicate DNA bands.

Of the three methods used, the optimized CNRG-CM method yielded favorable results in DNA extraction from all three soil types analyzed. On the other hand, using the commercial kit and the CTAB method, DNA could be extracted only from the SOX1 sample. The integrity of the DNA extracted with the three methods used in this study is shown in the agarose gels (Figure 3).

It can also be seen that, with the commercial kit and CTAB methods [28], DNA could be extracted only from the SOX1 sample, whereas the optimized CNRG-CM method extracted DNA from all three samples used in this work (Figure 2 and Figure 3). Differences in sample processing among the three methods may have affected the DNA extraction results.

### 4.3. DNA Quantification

The three soil samples treated with the CM-CNRG method yielded DNA quantifications ranging from 360 ng to 1300 ng/µL. However, these samples were co-extracted with contaminants, as reflected in the NanoDrop values (Table 2). In contrast to the other two methods tested in this work, the DNA quantification of the SOX 1 soil sample showed reduced values and was also co-extracted with contaminants.

DNA yield was normalized per gram of soil to account for differences in initial sample mass (CTAB-Wilson: 0.5 g; CNRG-CM: 1.0 g; commercial kit: 0.25 g). In Soils SC1 and SJ1, the CNRG-CM method was the only successful approach, achieving yields of 30.0 ± 0.6 µg g^−1^ and 48.0 ± 3.0 µg g^−1^, respectively, demonstrating excellent reproducibility. Both the CTAB-Wilson method and the commercial kit failed to recover detectable DNA in these samples. In Soil SOX1, all three methods were successful; the commercial kit achieved the highest yield of 43.2 ± 1.1 µg g^−1^, followed by CNRG-CM 39.0 ± 0.3 µg g^−1^; 90.3% relative efficiency, and Wilson 33.6 ± 0.6 µg g^−1^; 77.8% relative efficiency (Table 3).

## 5. Discussion

In the state of Chiapas, México, where sample SC1 was extracted, soil types such as Cambisol, Luvisol, Nitosol, and Andosol are abundant. Cambisol soils are characterized by their silty texture [33]. On the other hand, sample SJ1 was collected in the state of Jalisco, México, where, according to [34], at least five soil types are present in the municipality of Yahualica de González Gallo: Phaeozem, Cambisol, Luvisol, Planosol, and Lithosol. Luvisol is characterized by its clayey texture, and its color indicates the presence of iron oxides, which aligns with the description of sample SJ1. The soil sample SOX1 from the municipality of Pinotepa Nacional has a silty loam texture. According to [35], sandy and silty loam soils are found in the municipality of Pinotepa Nacional, which is consistent with the description in this study. It also has a dark color and high visible organic matter content [36].

Soil pH and color can influence nucleic acid extraction, leading to low or absent yields [37,38]. In soil SJ1, the pH is 4.8, and it has a reddish hue, according to [38]. Clays such as kaolinite and montmorillonite, as well as iron oxides, can adsorb DNA via positive surface charges, thereby reducing DNA extraction yield. In soil sample SC1, an acidic pH of 6 was measured, but the soil has a gray hue, according to [39]. The gray hue of soil is due to the presence of metals such as Al, Fe, and Mg. Some of these metals can bind to DNA, hindering DNA extraction.

Furthermore, soil composition influences pH, and metals like Al can degrade DNA or inhibit enzymatic activity [36]. Soil SOX1 has a slightly alkaline pH, high visible organic matter content, and a dark hue. With a silty loam texture, none of the three methods tested in this study had problems extracting DNA. From this, we infer that the presence of metals or clay that could interfere with nucleic acid extraction is low [32,40].

One of the primary differences among the three methods used in this study is that only the CNRG-CM method includes pretreatment with PBS and sodium phosphate washes before lysis.PBS is used as an isotonic solution to prevent cell lysis during the initial sample wash, which is crucial for preserving intact cells. Furthermore, PBS maintains a neutral pH (~7.4), inhibiting the activity of DNA-degrading nucleases and optimizing subsequent enzymatic reactions such as proteinase K. Part of the PBS pretreatment is to remove soluble salts, free organic matter, and contaminants that interfere with extraction, such as divalent ions and humates [41,42,43]. Subsequently, our optimized method includes two washes with sodium phosphate, which can act in soils with high clay content by functioning as an ion-exchange agent, displacing cations such as Fe^3+^, Al^3+^, and Ca^2+^ that bind DNA to minerals. This releases the electrostatically adsorbed DNA, improving its recovery [40]. In the study by [41], a DNA extraction method was standardized using agricultural soil. This method involved washing the soil with 1 M sodium phosphate to compete for cation binding to DNA, resulting in DNA free of cations and clay-DNA complexes. In another study, although the pretreatments were not identical to those used in this study, the principle of removing humic acids and PCR contaminants was the same. Ref. [38] used a pretreatment of polyvinylpolypyrrolidone (PVPP) and CaCO3, obtaining favorable results in DNA yield, even better than without pretreatment, on the same clay loam-sandy soil samples. This study suggests that pretreating soil samples can improve DNA extraction yields. Unlike our method, the Zymo Research kit uses a column to filter humic acids and organic matter after DNA resuspension, according to the manufacturer (Quick-DNA Fecal/Soil Microbe Miniprep Kit). However, no DNA extraction results were obtained. Studies such as that by [30] compared the effectiveness of two DNA extraction methods in a rhizosphere soil sample. One method used a commercial kit (Soil Master; EPICENTRE, Madison, WI, USA), while the other used pretreatment with PBS and PEG, followed by separation with organic solvents (phenol:chloroform:isoamyl). They found that using pretreatments such as PBS had a favorable effect on DNA purity and yield, whereas the commercial kit, although it achieved DNA extraction, produced DNA with higher impurities and a lower yield.

The ANOVA revealed significant differences among the three treatments used in this assay for nucleic acid extraction from the three soil samples with a *p* value < 0.05 and F value 18.29. The CNRG-CM method successfully extracted DNA from all three soil samples, whereas the CTAB-Wilson method and the commercial kit yielded DNA only from the SOX1 sample. In this study, the CNRG-CM-treated samples were quantified and found to contain impurities (Table 2). This may be due to the persistent presence of humic acids and organic solvents present during DNA extraction [44,45]. RNase treatment could be incorporated to obtain cleaner DNA samples; however, our objective was to demonstrate the DNA as it emerges directly from the extraction process, without additional purification steps. However, considering that the amplification of the 16S rRNA gene from soil samples SC1, SOX1, and SJ1 treated with the CNRG-CM method was favorable, obtaining an expected fragment of 1500 bp without the addition of any PCR stabilizer such as bovine serum albumin (BSA), glycerol, or DMSO, we consider that the presence of these contaminants does not represent a serious problem in the DNA sample obtained in this assay, unlike other published methods that do use albumin or at least recommend its use [46,47].

Regarding the SJ1 and SC1 samples processed with the CTAB-Wilson and commercial kit methods, it is important to clarify that NanoDrop quantification did detect nucleic acid readings; however, these exhibited very low A260/A230 and A260/A280 ratios, indicating significant contamination with humic acids, phenolic compounds, and proteins. Furthermore, no discrete genomic DNA band was observed in the agarose gel electrophoresis for these samples. The faint bands visible in the gel likely correspond to co-extracted RNA or degraded nucleic acids rather than intact genomic DNA. Consequently, these samples were considered negative for DNA recovery (Table 2 and Table 3), as the extracted material did not meet the quality thresholds required for downstream molecular applications [48].

The CNRG-CM method consistently outperformed both the Wilson protocol and commercial kit in complex soil types (SC1 and SC2), achieving yields of 30–48 µg g^−1^ soil, values that fall within the typical range reported for optimized soil DNA extraction protocols (12–60 µg g^−1^) [49,50]. This performance aligns with previous comparisons demonstrating that custom protocols using larger input masses (≥1 g) better overcome matrix interferences such as humic acids and clay minerals, common in agricultural soils [51]. The commercial kit, while achieving the highest yield per gram in Soil SOX1 (43.2 µg g^−1^), exhibited failure in Soils SC1 and SC2, confirming previous findings that such kits are optimized for favorable matrices but lack robustness in heterogeneous samples [52,53].

Finally, we emphasize that our optimized method, in addition to being optimal for challenging soils, is also more economical than commercial kits and other phenol:chloroform-based methods. This is because our method does not use proteases such as proteinase K or lysozyme, which, although they have proven very efficient for enzyme degradation and DNA release, we have shown to be dispensable. This is due to the beating with glass beads we use, which has proven very efficient for membrane disruption [54,55,56,57]. Alternatively, the addition of beta-mercaptoethanol to lysis buffers in commercial kits such as the one used in this study [58,59], in addition to the complete absence of phenol in the DNA extraction process, as specified in several nucleic acid extraction protocols [60].

## 6. Conclusions

The CNRG-CM method developed in this study enables efficient extraction of high-quality metagenomic DNA from three types of agricultural soils with distinct physicochemical properties: Amatán, Chiapas (SC1: silt loam, pH 6.0), Yahualica de González Gallo, Jalisco (SJ1: clay loam, pH 4.8), and Pinotepa Nacional, Oaxaca (SOX1: silt loam, pH 7.6). This method achieved DNA yields of 30.0 ± 0.6 µg g^−1^ and 48.0 ± 3.0 µg g^−1^ in the challenging soils SC1 and SJ1, respectively, where both the traditional CTAB-Wilson method and commercial kit failed completely (0 µg g^−1^). In the more favorable Soil SOX1, CNRG-CM yielded 39.0 ± 0.3 µg g^−1^, demonstrating competitive performance with the commercial kit (43.2 ± 1.1 µg g^−1^). Extracted DNA concentrations ranged from 1000 to 1600 ng/μL, and successful amplification of the 16S rRNA gene was achieved without PCR stabilizers. Compared to the traditional CTAB-Wilson method and commercial kits, the CNRG-CM method exhibits superior robustness across diverse soil matrices, broader applicability to complex soils, and significantly lower cost, providing a reliable DNA extraction protocol for metagenomic studies of agricultural soils.

## Figures and Tables

**Table 1:** Soil sample characteristics.

Sample	Soil Textura	pH	Color	Organic Matter
SC1	Franco-limous	6	Opaque gray	Low organic matter.
SJ1	Clayey Franco	4.8	Reddish-brown	Medium organic matter.
SOX1	Franco-limous	7.6	Dark	High organic matter content.

**Table 2:** Quantification with a nanodrop of DNA extracted using three methods.

Sample	CTAB Method	A260/230	A260/280	Commercial Kit Method	A260/230	A260/280	CNRG-CM Method	A260/230	A260/280
**SC1 ng/uL**	N/D	N/D	N/D	N/D	N/D	N/D	1000 ng/uL	1.60	1.50
**SJ1 ng/uL**	N/D	N/D	N/D	N/D	N/D	N/D	1600 ng/uL	1.75	1.77
**SOX1 ng/uL**	560 ng/uL	1.70	1.72	360 ng/uL	1.80	1.80	1300 ng/uL	1.70	1.70

**Table 3:** DNA yield and percentage efficiency of three soil samples with three replicates.

Soil	Method	Mean Concentration (ng/µL) ± SD	DNA Yield (µg g^−1^) ± SD	Efficiency (%)
SC1	CTAB-Wilson	0.0 ± 0.0	0.00 ± 0.00	0.0
SC1	CNRG-CM	1000.0 ± 20.0	30.00 ± 0.60	100.0
SC1	Kit-commercial	0.0 ± 0.0	0.00 ± 0.00	0.0
SJ1	CTAB-Wilson	0.0 ± 0.0	0.00 ± 0.00	0.0
SJ1	CNRG-CM-	1600.0 ± 100.0	48.00 ± 3.00	100.0
SJ1	Kit-commercial	0.0 ± 0.0	0.00 ± 0.00	0.0
SOX1	CTAB-Wilson	560.0 ± 10.0	33.60 ± 0.60	77.8
SOX1	CNRG-CM	1300.0 ± 8.7	39.00 ± 0.26	90.3
SOX1	Kit-commercial	360.0 ± 8.9	43.20 ± 1.07	100.0

## Data Availability

The original contributions presented in this study are included in the article. Further inquiries can be directed to the corresponding author.
